# *Nocardia brasiliensis* endophthalmitis initially misdiagnosed as uveitis: a case report

**DOI:** 10.1186/s12348-026-00602-0

**Published:** 2026-06-23

**Authors:** Xue Zhang, Liping Du, Xuemin Jin, Jingjing Sun, Guangqi An, Lin Li, Peizeng Yang, Fuzhen Li

**Affiliations:** 1 Department of Ophthalmology, The First Affiliated Hospital of Zhengzhou University, Henan Province Eye Hospital, Henan International Joint Research Laboratory for Ocular Immunology and Retinal Injury Repair, Henan Province Engineering Research Center of Fundus Disease and Ocular Trauma Prevention and Treatment, Zhengzhou, 450052 China; 2 Bascom Palmer Eye Institute, University of Miami Miller School of Medicine, Miami, FL USA; 3 Department of Clinical Laboratory, The First Affiliated Hospital of Zhengzhou University, Zhengzhou, China; 4 Henan Provincial People’s Hospital, Zhengzhou University People’s Hospital, Henan Eye Hospital, Zhengzhou, China; 5 Ophthalmology Medical Center, The First Affiliated Hospital of Chongqing Medical University, Chongqing Key Laboratory for the Prevention and Treatment of Major Blinding Eye Diseases, Chongqing Branch (Municipality Division) of National Clinical Research Centre for Ocular Diseases, Chongqing, China

**Keywords:** *Nocardia brasiliensis*, Endophthalmitis, Metagenomic next-generation sequencing, Uveitis

## Abstract

**Background:**

Endophthalmitis caused by *Nocardia brasiliensis* is extremely rare and typically affects immunocompromised individuals, frequently leading to severe vision loss due to diagnostic delays. We report a case of *N. brasiliensis* endophthalmitis in an older man without prior history of systemic immunosuppression but with newly identified diabetes mellitus, characterized by an indolent initial course followed by fulminant progression.

**Case presentation:**

A 67-year-old man without known systemic immunosuppression presented with a two-month history of recurrent right-eye pain and redness, followed by rapid vision loss and a hypopyon. Aqueous humor analysis and metagenomic sequencing identified *N. brasiliensis*. Despite intravitreal amikacin, systemic antimicrobial therapy, and subsequent pars plana vitrectomy with silicone oil tamponade, intraocular inflammation advanced, resulting in worsening corneal opacification, irreversible structural damage, and a final best-corrected visual acuity of light perception.

**Conclusions:**

N. brasiliensis endophthalmitis may progress rapidly and result in severe, irreversible ocular damage, even in patients without overt systemic immunodeficiency. Early microbiologic identification and prompt, targeted antimicrobial therapy combined with timely surgical intervention are critical, although visual outcomes may remain poor in advanced cases.

## Background

*Nocardia* species are strictly aerobic, Gram-positive or Gram-variable, and weakly acid-fast bacilli ubiquitous in soil, decaying matter, and organic water [1, 2]. As opportunistic pathogens, they typically cause acute or chronic suppurative and granulomatous infections, often entering through the respiratory tract or skin wounds, with immunocompromised individuals being the most susceptible [1, 2]. Pulmonary infections are the most common clinical manifestation, with *Nocardia asteroides* accounting for over 70% of human nocardiosis [2]. In contrast, *Nocardia brasiliensis* most commonly causes cutaneous disease, and ocular involvement is rare [2, 3]. Accurate species identification is essential for guiding appropriate treatment and avoiding poor outcomes. However, non-specific clinical features and low awareness often lead to misdiagnosis.

Here, we report a case of *N. brasiliensis* endophthalmitis notable for its prolonged indolent course followed by rapid clinical deterioration, underscoring the diagnostic and therapeutic challenges posed by this pathogen in ocular settings.

## Case presentation

### Initial presentation

**Fig. 1:**
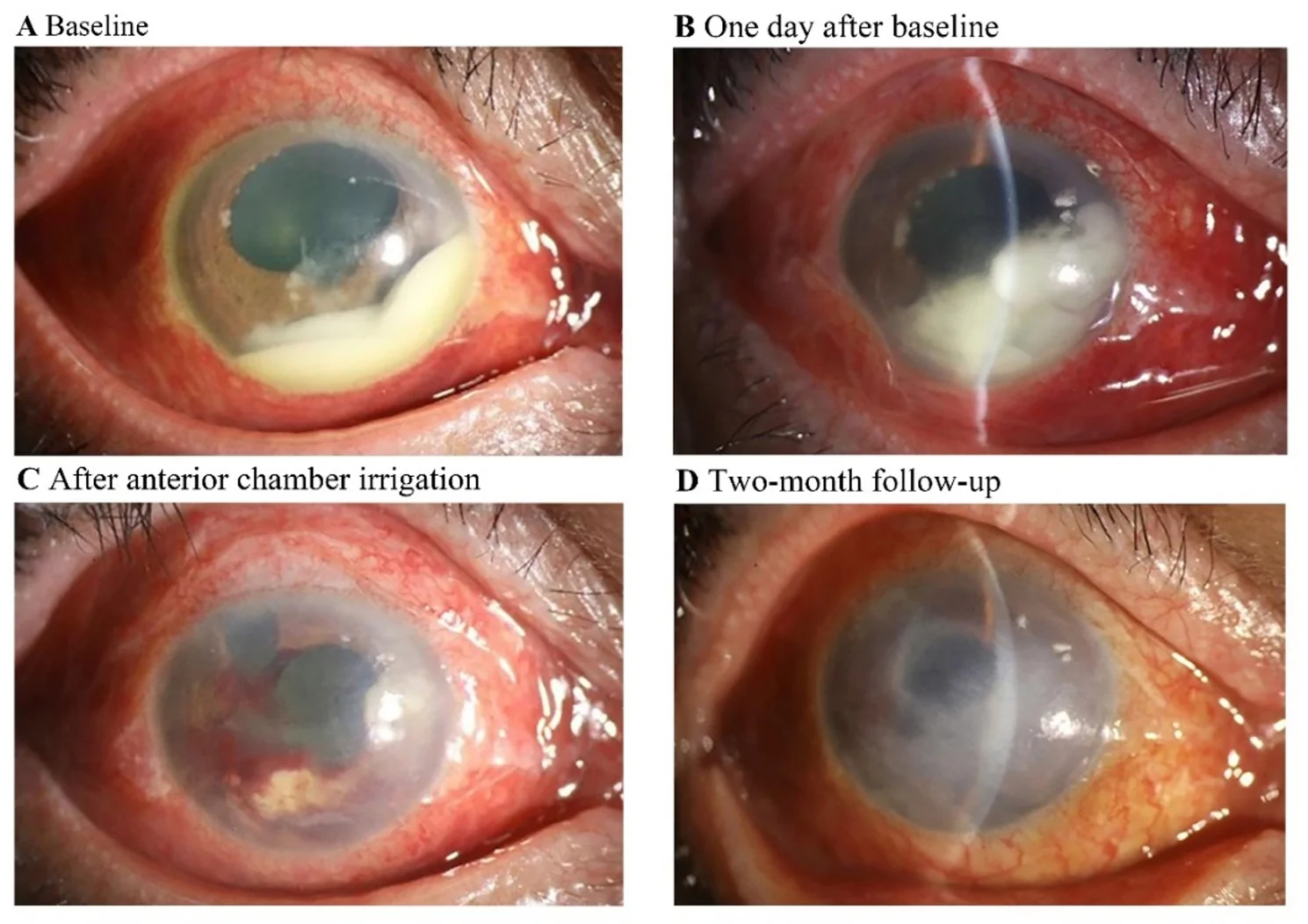
Clinical course in a 67-year-old man with *Nocardia brasiliensis* endophthalmitis. (**A**) Baseline image shows a shallow anterior chamber with a 2-mm hypopyon, keratic precipitates, and marked anterior chamber flare. (**B**) One day after baseline, the hypopyon enlarged to 4 mm with increased inflammation. (**C**) After anterior chamber irrigation, part of the hypopyon was removed, and intravitreal amikacin was administered. (**D**) At 2 months, the eye shows corneal opacity and irreversible structural damage

**Fig. 2:**
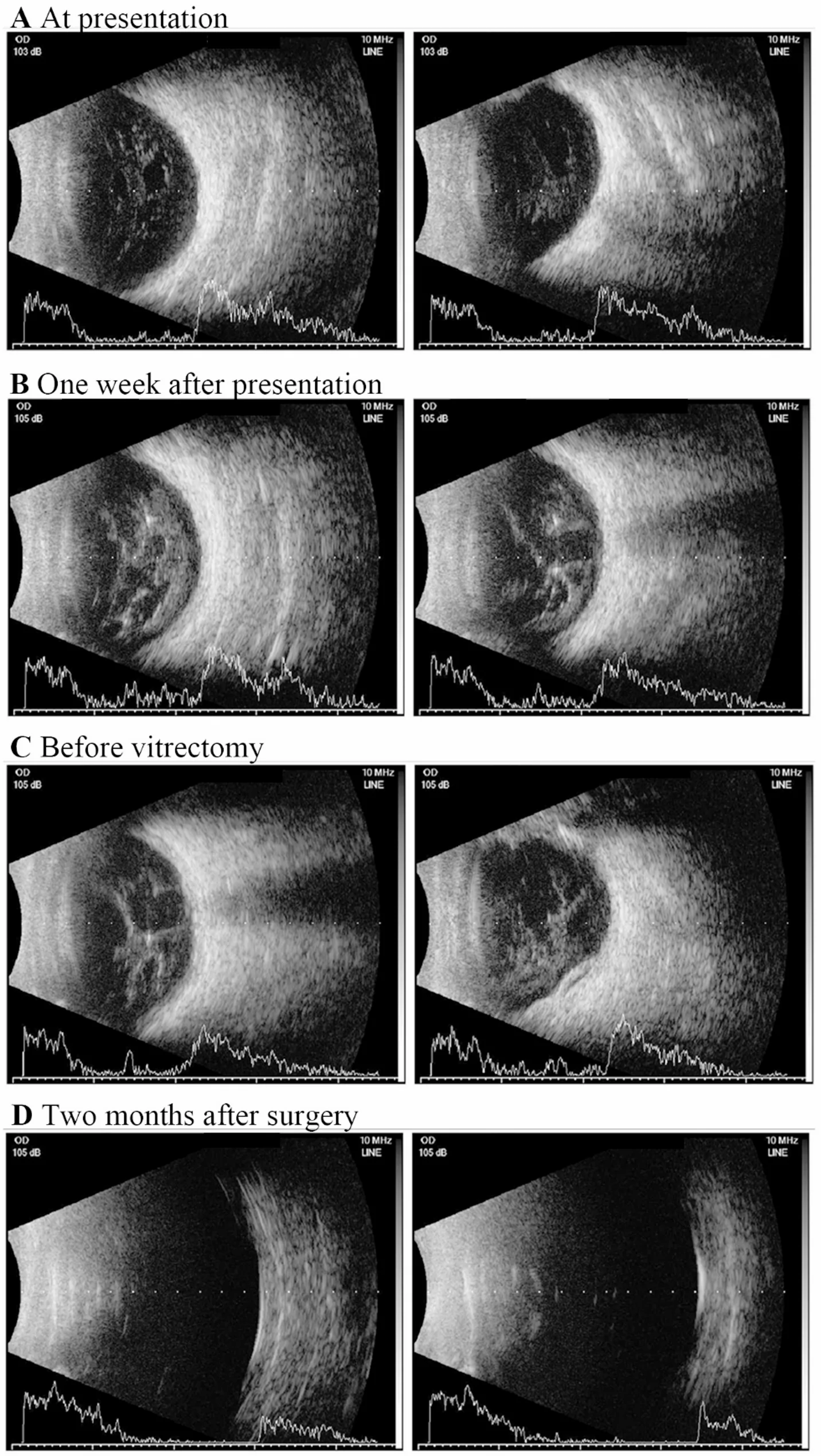
Serial B-scan ultrasonography of the right eye during follow-up. (**A**) At presentation, B-scan ultrasonography showed mild vitreous opacities, with no evident retinal detachment, choroidal detachment, or intraocular mass. (**B**) One week after presentation, repeat B-scan ultrasonography demonstrated increased vitreous echoes and debris. (**C**) Before vitrectomy, B-scan ultrasonography showed further progression, including dense vitreous opacities, localized shallow retinal detachment, and thickened scleral echoes. (**D**) Two months after surgery, B-scan ultrasonography showed typical postoperative features following vitrectomy and silicone oil tamponade

A 67-year-old man without known systemic immunosuppression presented with a two-month history of recurrent right eye pain, redness, and episodic intraocular inflammation. The patient was a farmer and denied any history of ocular trauma or previously diagnosed systemic disease at presentation. However, laboratory workup revealed a glycated hemoglobin (HbA1c) level of 7.0%, indicating previously unrecognized diabetes. He had previously been evaluated at a local hospital and was diagnosed with right eye uveitis. After receiving topical and systemic corticosteroid therapy, his symptoms improved but recurred repeatedly. Two days before admission, his vision rapidly declined, and a 2-mm hypopyon developed (Fig. 1A). On examination, there was no evidence of ocular trauma or any surgical entry site. Posterior segment visualization was limited by media opacity. Initial B-scan ultrasonography showed mild vitreous opacities, with no evident retinal detachment, choroidal detachment, or intraocular mass (Fig. 2A). Systemic evaluation, including inflammatory markers (normal C-reactive protein and procalcitonin levels) and imaging studies (abdominal ultrasonography, chest computed tomography, and brain magnetic resonance imaging), did not identify any clinical signs of infection. The portal of entry for the infection remained unclear. No definite exogenous entry site was identified, although an unrecognized minor ocular inoculation could not be excluded.

### Initial management and diagnostic testing

**Fig. 3:**
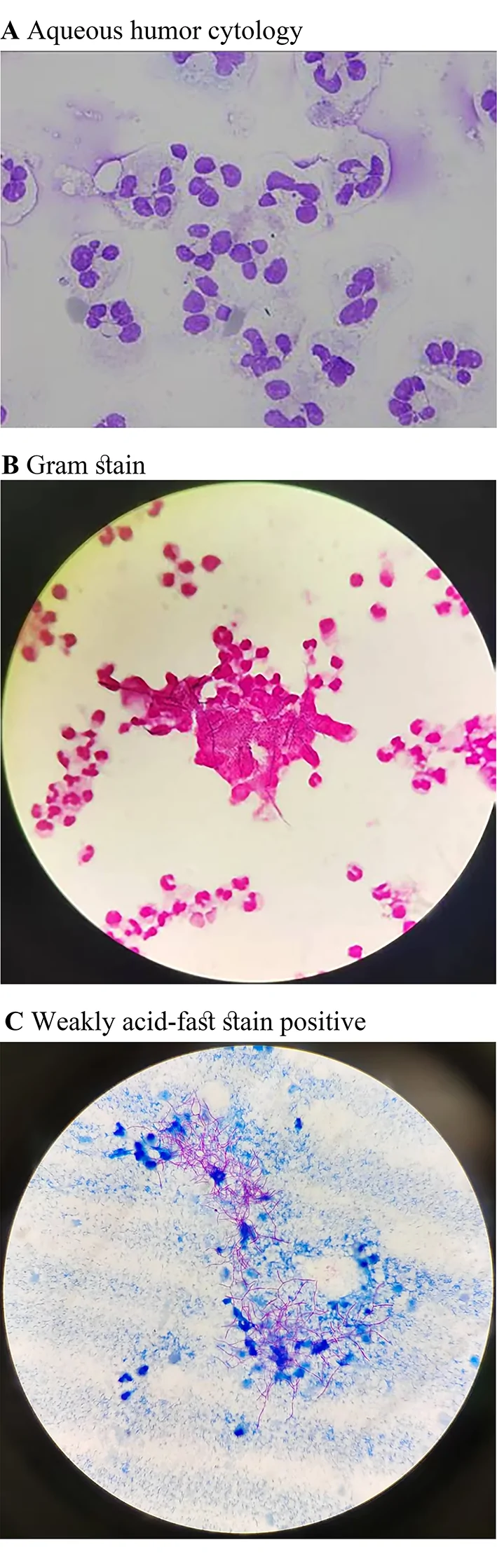
Microbiological and cytologic analysis of aqueous humor. (**A**) Cytologic analysis of aqueous humor shows 95% neutrophils (original magnification ×100). (**B**) Gram stain reveals slender, filamentous, branching Gram-positive bacilli. (**C**) Modified acid-fast staining shows weak positivity with the same filamentous, branching morphology

Diagnostic anterior chamber paracentesis was performed; at that time, the anterior chamber was markedly shallow, and the hypopyon had increased to 4 mm compared with the previous day (Fig. 1B). Cytologic analysis of the aqueous humor showed that 95% of the cells were neutrophils (Fig. 3A). Gram and modified acid-fast stains demonstrated slender, filamentous, branching Gram-positive bacilli (Fig. 3B and C).

Metagenomic next-generation sequencing (mNGS) and bacterial culture and identification successively confirmed *N. brasiliensis.* Susceptibility testing confirmed amikacin sensitivity (Table 1). The patient underwent anterior chamber irrigation and received an intravitreal amikacin injection (0.4 mg). Part of the hypopyon was removed during the procedure (Fig. 1C). After surgery, his best-corrected visual acuity (BCVA) was 20/100. The patient was then treated with an intensive antimicrobial regimen. Topical therapy included amikacin drops (hourly), tobramycin drops (every 4 h), gatifloxacin gel (every 12 h), brimonidine tartrate drops (twice daily), carteolol hydrochloride drops (twice daily), and pranoprofen drops (4 times daily). Systemic therapy consisted of intravenous piperacillin–tazobactam (4.5 g every 6 h for 4 days) combined with oral trimethoprim-sulfamethoxazole (TMP-SMX; 320 mg trimethoprim/1600 mg sulfamethoxazole per dose, three times daily; total daily dose, 960 mg/4800 mg). One week after presentation, repeat B-scan ultrasonography demonstrated significantly increased vitreous echoes and debris (Fig. 2B). The patient was subsequently discharged and continued treatment at another hospital.

**Table 1:** Antibiotic susceptibility results

Antibiotic	Abbreviation	MIC (µg/mL)	Interpretation	Breakpoints (≤ S/≥*R*, µg/mL)
Amikacin	AMK	1	Sensitive	≤ 8; ≥16
Amoxicillin/clavulanic acid	AMC	16	Intermediate	≤ 8/4; ≥32/16
Ceftriaxone	CRO	32	Intermediate	≤ 8; ≥64
Ciprofloxacin	CIP	2	Intermediate	≤ 1; ≥4
Clarithromycin	CLR	1	Sensitive	≤ 2; ≥8
Imipenem	IPM	64	Resistant	≤ 4; ≥16
Linezolid	LNZ	1	Sensitive	≤ 8
Minocycline	MNO	2	Intermediate	≤ 1; ≥8
Moxifloxacin	MFX	0.5	Sensitive	≤ 1; ≥4
Trimethoprim/sulfamethoxazole	SXT	0.25	Sensitive	≤ 2/38; ≥4/76
Tobramycin	TOB	4	Sensitive	≤ 4; ≥16
Cefepime	FEP	32	Resistant	≤ 8; ≥32
Cefotaxime	CTX	32	Intermediate	≤ 8; ≥64

Abbreviations: MIC, minimum inhibitory concentration; S, susceptible; I, intermediate; R, resistant

### Progression and surgical management

At that facility, the existing regimen was continued, with the addition of compounded topical tacrolimus (1 mg mixed with 0.3% sodium hyaluronate eye drops, once daily) and topical voriconazole (50 mg dissolved in 10 mL sterile water for injection, twice daily). The remainder of the previous regimen was continued unchanged. Despite these interventions, within one week of the initial intravitreal amikacin injection, his vision declined to counting fingers. Repeat B-scan ultrasonography showed increased vitreous debris, localized shallow retinal detachment, and thickened scleral echoes (Fig. 2C). The patient underwent pars plana vitrectomy with silicone oil tamponade, phacoemulsification, and intraocular lens implantation. Intraoperatively, yellow purulent material was observed beneath the macula and around the optic disc, with preretinal hemorrhage. The intraocular lens was implanted after careful intraoperative consideration to help limit silicone oil migration into the anterior chamber and reduce the risk of secondary anterior segment complications, particularly intraocular pressure elevation.

### Follow-up and outcome assessment

Over a two-month follow-up period, the infection continued to progress despite aggressive treatment. BCVA declined to light perception, with progressive corneal opacification leading to irreversible structural damage and complete loss of vision in the affected eye (Fig. 1D). B-scan ultrasonography displayed typical features following silicone oil tamponade (Fig. 2D). No obvious treatment-related adverse events were documented during follow-up. Regarding the patient’s newly diagnosed diabetes mellitus, glycemic control improved with dietary modification alone, and follow-up testing showed an HbA1c level of 5.9%.

## Discussion

Nocardia endophthalmitis is an uncommon but serious intraocular infection that usually follows ocular trauma or surgery [4]. Nocardia species typically cause keratitis, whereas intraocular extension is rare [3, 4]. N. brasiliensis usually produces cutaneous disease and has rarely been reported in the eye [3]. Our case was unusual because no history of ocular trauma or surgery was identified. However, given the patient’s occupation as a farmer, with frequent exposure to soil and organic material, an exogenous source of inoculation remains plausible, particularly through minor and unrecognized ocular microtrauma. Although the patient had no known systemic immunosuppression, newly identified diabetes mellitus (HbA1c 7.0%) may have increased susceptibility to infection, as diabetes is associated with impaired host defense and greater vulnerability to infection [5], and may have contributed to susceptibility in this case. Furthermore, although no overt systemic immunodeficiency was identified at presentation, long-term follow-up remains important. Any recurrent or additional opportunistic infection should prompt further evaluation for a possible underlying immunologic abnormality not detected during the initial workup. Early symptoms (ocular pain, conjunctival injection, and decreased vision) are nonspecific and may delay diagnosis. In our patient, Gram staining and mNGS rapidly identified the organism and helped guide therapy.

TMP-SMX has long been considered a cornerstone of therapy for Nocardia infection [2]. In the study by Kannan et al., Nocardia isolates demonstrated the highest susceptibility to amikacin (100%), followed by gatifloxacin (82.1%), levofloxacin (71.4%), ofloxacin (71.4%), and moxifloxacin (67.9%). Overall susceptibility to fluoroquinolones was 82.2%. In contrast, the highest resistance rates were observed against cefazolin and ceftazidime (96.4%), followed by vancomycin (71.4%) [4]. These findings are consistent with those reported by Haripriya et al. [6] and align with the antimicrobial susceptibility testing results of our patient (Table 1).

The poor visual outcome in this case was likely multifactorial. First, the initial misdiagnosis of uveitis led to a two-month delay in appropriate antimicrobial therapy. During this period, the patient was treated with topical and systemic corticosteroids. Although corticosteroids may have temporarily reduced ocular inflammation, they may also have masked the underlying infectious process and impaired local host defense, thereby permitting continued intraocular progression. In the context of Nocardia infection, this delay likely allowed ongoing microbial proliferation and resulted in extensive irreversible structural damage before targeted therapy was initiated. Second, despite prompt intravitreal amikacin once susceptibility results became available, the disease continued to worsen despite intensive topical and systemic antimicrobial therapy. In this setting, repeated intravitreal injection alone was unlikely to provide adequate infection control. The rapid structural deterioration, together with the intraoperative finding of purulent material beneath the macula and around the optic disc, suggested that part of the infectious burden had become sequestered in compartments with limited antimicrobial penetration. Moreover, the granulomatous nature of Nocardia infection may have further limited drug access to the lesion, making surgical clearance necessary [2, 7].

The surgical management of this case required careful intraoperative judgment regarding intraocular lens (IOL) implantation. We recognize that implantation of any intraocular prosthetic material in an actively infected eye carries a theoretical risk of bacterial sequestration and biofilm formation, which may compromise infection control [8, 9]. Alternative approaches to reduce anterior migration of silicone oil were also considered, including leaving the eye aphakic or using a mechanical barrier, such as silicone oil retention sutures [10, 11]. In this case, however, the eye had already sustained severe structural damage, and the visual prognosis was judged to be very poor at the time of surgery. The eye was also expected to remain silicone oil dependent for a prolonged period. In this setting, IOL implantation was considered a practical means of partial compartmentalization to help limit anterior migration of silicone oil and reduce secondary anterior segment complications, particularly intraocular pressure elevation. This decision reflected a careful balance between the potential infection-related risks of prosthetic implantation and the need for structural stabilization in a fulminant, severely damaged eye.

Ultimately, severe structural damage markedly limited both infection control and visual recovery. This case also highlights the importance of close collaboration between ophthalmologists and microbiologists, as prompt microbiologic evaluation enabled early pathogen identification and guided treatment selection.

## Limitations

This report describes a single case, which limits generalizability. The absence of an identifiable portal of entry and the lack of serial quantitative microbiologic data preclude definitive conclusions regarding disease origin and treatment response. Longer follow-up and additional cases are needed to better define prognosis and optimal management.

## Conclusion

This case underscores the severe clinical course and poor visual prognosis of N. brasiliensis endophthalmitis. Early manifestations frequently mimic uveitis, and prior corticosteroid exposure can obscure the infectious process, leading to delayed diagnosis and rapid structural damage. Consequently, early microbiologic evaluation with Gram staining and mNGS is critical. Clinicians should maintain a high index of suspicion for Nocardia infection in patients with steroid-refractory intraocular inflammation, especially in those with a history of soil exposure, and avoid further corticosteroid escalation until infection has been reasonably excluded.

## Data Availability

No datasets were generated or analysed during the current study.

## Abbreviations

BCVA: Best-corrected visual acuity
HbA1c: Glycated hemoglobin
mNGS: Metagenomic next-generation sequencing
TMP-SMX: Trimethoprim-sulfamethoxazole
IOL: Intraocular lens

## Declarations

### Image permissions and credits

All figures in this manuscript were generated by the authors and are original. No images were reproduced or adapted from previously published sources, and therefore no additional permissions are required.
